# Correction to “Potential of Circulating lncRNA CASC2 as a Biomarker in Reflecting the Inflammatory Cytokines, Multi‐Organ Dysfunction, Disease Severity, and Mortality Insepsis Patients”

**DOI:** 10.1002/jcla.70168

**Published:** 2026-01-27

Wang R, Zhao J, Wei Q, et al. Potential of circulating lncRNA CASC2 as a biomarker in reflecting the inflammatory cytokines, multi‐organ dysfunction, disease severity, and mortality in sepsis patients. *J Clin Lab Anal*. 2022;36:e24569. https://onlinelibrary.wiley.com/doi/epdf/10.1002/jcla.24569.

Due to differing institutional requirements regarding the designation of corresponding authors, the affiliation of Qingchun Dai^1^ designates the corresponding author position at the end of the list, whereas the affiliation of Cuicui Liu^2^ requires it to be placed at the beginning. After comprehensive discussion and mutual agreement among all co‐authors, it has been collectively decided to revise the author order by changing the positions of these two corresponding authors.

The updated authorship sequence is shown below:

Rui Wang^1^ | Jinglin Zhao^1^ | Qi Wei^1^ | Hao Wang^1^ | Chao Zhao^2^ | Caihong Hu^2^ | Yu Han^1^ | Zhi Hui^1^ | Long Yang^1^ | Cuicui Liu^2^ | Qingchun Dai^1^.

We apologize for this error.

